# Nucleic Acid-Loaded Lipid Nanoparticle Interactions with Model Endosomal Membranes

**DOI:** 10.1021/acsami.2c06065

**Published:** 2022-06-27

**Authors:** Alice Spadea, Mark Jackman, Lili Cui, Sara Pereira, M. Jayne Lawrence, Richard A. Campbell, Marianne Ashford

**Affiliations:** † NorthWest Centre for Advanced Drug Delivery (NoWCADD), School of Health Sciences, University of Manchester, Oxford Road, Manchester M13 9PT, U.K.; ‡ Division of Pharmacy and Optometry, Faculty of Biology, Medicine and Health, University of Manchester, Oxford Road, Manchester M13 9PT, U.K.; ∥ Advanced Drug Delivery, Pharmaceutical Sciences, AstraZeneca R&D, Cambridge CB2 0AA, U.K.; § Advanced Drug Delivery, Pharmaceutical Sciences, AstraZeneca R&D, Macclesfield SK10 2NA, U.K.

**Keywords:** lipid nanoparticles, endosomal escape, Langmuir trough, ellipsometry, Brewster angle microscopy (BAM)

## Abstract

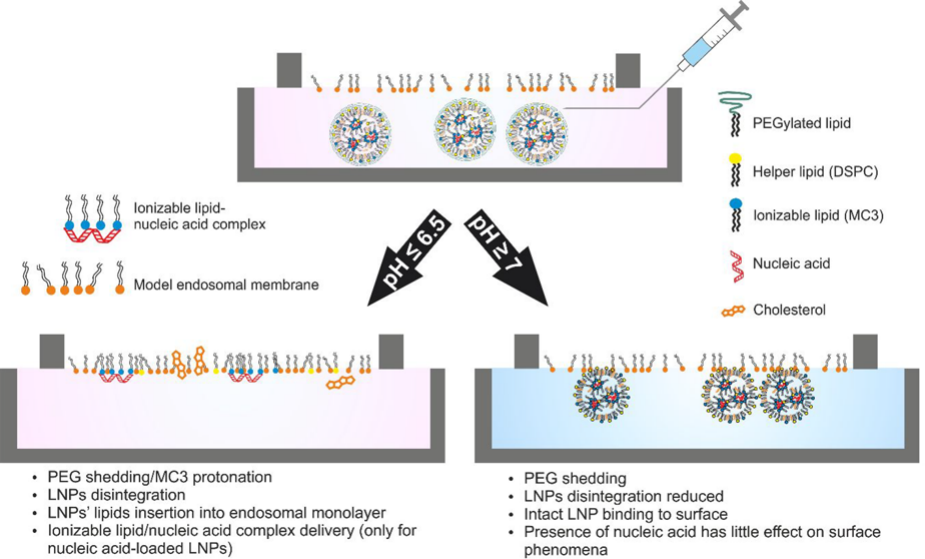

Lipid nanoparticles (LNPs) are important delivery systems for RNA-based therapeutics, yet the mechanism of their interaction with endosomal membranes remains unclear. Here, the interactions of nucleic acid-loaded LNPs that contain an ionizable lipid with models of the early and late endosomal membranes are studied, for the first time, using different reflectometry techniques. Novel insight is provided with respect to the subphase pH, the stage of the endosome, and the nature of the nucleic acid cargo. It is found that the insertion of lipids from the LNPs into the model membrane is greatest at pH 6.5 and 5.5, whereas at higher pH, lipid insertion is suppressed with evidence instead for the binding of intact LNPs, demonstrating the importance of the pH in the fusion of LNPs undergoing the endosomal pathway. Furthermore, and independently of the pH, the effect of the early- versus late-stage endosomal models is minimal, suggesting that the increased fluidity and anionic nature of the late endosome has little effect on the extent of LNP interaction. Last, there is greater nucleic acid delivery from LNPs containing mRNA than Poly(A), indicating that the extent of interaction can be tuned according to the nature of the nucleic acid cargo. Such new information on the relative impact of factors influencing nucleic acid delivery by LNP interactions with endosomal membranes is important in the design and tuning of vehicles with improved nucleic acid delivery capacities.

## Introduction

RNA delivery and gene therapy hold great potential for the treatment of many diseases, for example, expression of a required protein (mRNA delivery) or gene knockdown (siRNA delivery). Lipid nanoparticles (LNPs) are considered to be the most successful carriers developed for RNA delivery, particularly of siRNA,^1^ with Patisiran being the first siRNA-based medicine using LNPs to target the delivery of siRNA to the liver.^2^ The use of LNPs has now been extended to contain larger mRNA to prepare vaccines against infectious diseases such as COVID-19. Indeed, the first two COVID-19 vaccines to report the results of their clinical trials are mRNA-formulated LNPs.^3^

The realization of RNA therapeutics in the clinic has been largely hindered over the years because of problems with the stability of nucleic acids in biological fluids, the lack of cell “targetability”, immunogenicity, and, as a result of its large size and hydrophilic and anionic nature, its very poor membrane permeability.^4^ As a consequence, many research efforts in recent years have focused on the development of nanoparticulate carrier systems that are efficiently taken up by the cell, escaping the endosomes to deliver their RNA payload intact to the cytosol.^5−7^ To achieve this, the carrier system must successfully protect its RNA cargo during transportation to the target cells and, upon its passage through the endosome, must release the intact RNA into the cytoplasm. However, the efficient escape of the carrier and/or RNA from the intracellular endosomal/lysosomal compartments remains a major challenge.

Endosomes are internal cellular vesicles that originate from the invagination of the plasma membrane, constituting an important part of the intracellular degradation pathway.^8^ The vesicles that originate from the internalization of material by a cell, which are spherical in shape and possess diameters of about 400–500 nm, undergo a maturation process from early endosome to multivesicular bodies, containing intraluminal vesicles, to late endosome.^9,10^ There is evidence that endosomes at different stages of the degradation pathway exhibit differences in the protein and lipid compositions of their membrane^11^ and internal pH. For example, the internal membrane of the late endosome contains the anionic polyunsaturated lipid bis(monoleoylglycero)phosphate [BMP]. [BMP is sometimes called lysobisphosphatidic acid; this term is incorrect because the bis prefix suggests the linking together of two such phosphatidic acids with one or two fatty acid residues in positions 2 and 3 of the glycerol moiety; however, the compound is two monoacylglycerols bound to a phosphate group.^23^] BMP is a lipid that is unique to to the late endosome,^12^ and the early endosomes exhibit a pH of 6.8–5.9^13^ and the late endosomes a lower pH of 5.5–5.0.^14^ The endosomal escape is a crucial stage in the effective delivery of intact RNA to avoid being digested in the lysosomes, the structure into which the late endosome matures.^15^ However, the process by which endosomal escape occurs is poorly understood, with <2% of siRNA administered in LNPs being estimated to escape the endosomes to reach the cytosol.^16,17^

Recently, it has been reported that ionizable aminolipids, such as heptatriaconta-6,9,28,31-tetraen-19-yl 4-(dimethylamino)butanoate (DLin-MC3-DMA or MC3), when present in LNPs in combination with other lipids including the phospholipid distearoylphosphatidylcholine (DSPC), cholesterol, and the PEGylated lipid DMG-PEG-2000, make LNPs more effective at delivering functional mRNA to cytosol of the cells compared to DLin-MC3-DMA-deprived LNPs.^7^ Research has shown that the use of ionizable cationic lipids with an apparent p*K*_a_ of 6.44 in the preparation of LNPs^18^ allows the particles to be formulated at low pH and to maintain a neutral or low cationic surface charge density at pH 7.4, enabling better circulation properties.^19^

It is proposed that the core of the LNP contains all of the RNA (either siRNA or mRNA), thereby protecting it, most or all of the ionizable cationic lipid, and some of the cholesterol, and is surrounded by a layer of the more polar DSPC, the PEGylated lipid, and the remaining cholesterol.^20−22^ Shedding of the PEGylated lipid from the exterior of the particle is considered to be a requirement for internalization of the LNP into the cell.^20^ Once within the low-pH environment of the endosome, the ionized cationic lipids of the LNPs are thought to interact with the anionic lipids present in the endosomal membrane, resulting in the formation of hexagonal structures that can disrupt the membrane, allowing endosomal escape.^24^

Complementary to investigations of LNP efficacy *in vitro* and *in vivo*,^25,26^ biophysical studies using a range of model cell membranes (including phospholipid vesicles and planar bilayers and monolayers) as experimental platforms can provide insight into the mechanistic nature of LNP interactions.^27^ Although lipid monolayers prepared at the air/water interface on a Langmuir trough lack the two lipid leaflets inherent to cell membranes, they are widely considered to be informative models for interaction studies involving drugs or drug carriers^28,29^ because they can be used to mimic membrane fluidity by controlling the surface pressure of the monolayer.^30^

In the present study, Langmuir monolayers are used as a platform to study the interactions of LNPs with models of early- and late-stage endosomal membranes. The LNPs, without cargo or containing either Firefly luciferase (FLuc) mRNA or the adenosine monophosphate homopolymer poly(adenylic acid) [Poly(A)], are prepared using a standard microfluidic mixing procedure and comprise DLin-MC3-DMA, DSPC, cholesterol, and DMG-PEG-2000 in the respective molar ratio 50:10:38.5:1.5.^31^ Models of the early endosomal membrane (EEM) and late endosomal membrane (LEM) each comprise a mixture of four lipids to mimic compositions found *in vivo*.^23,32^ As validation of the experimental approach, it is worth noting that Langmuir monolayers are planar and endosomal membranes are curved. The curvature of the endosome varies according to its size and is generally much less than the curvature of the LNPs (size <100 nm).^33^ As a result, the LNPs experience a low effective curvature of the endosome, and, hence, we consider planar model membranes to be a valid experimental platform. In addition to monitoring the changes in surface pressure that occur in the presence of LNPs, two *in situ* optical reflectometry techniques are applied here to provide further information about the interaction mechanism. Ellipsometry is used to measure the change in the polarization of light upon reflection at the interface on the second time scale,^34^ which can be related to the extent of interactions,^35^ as well as the presence of lateral inhomogeneities.^36^ Brewster angle microscopy (BAM) also exploits the reflection of polarized light at the interface but is used to image the lateral morphology on a micrometer scale,^37^ which can be related to the presence of lipid domains of different phases in the monolayer^38^ or extended structures in contact with it.^39^ The combination of surface pressure and ellipsometry data can be beneficial to distinguishing different types of processes, such as the insertion of lipid, binding of LNPs, and delivery of ionizable lipid–nucleic acid complexes. In fact, the surface pressure is most sensitive to the packing of lipid molecules in the monolayer and ellipsometry to the total amount of interfacial material per unit area and its ordering, while BAM can provide its own insight or support for the data interpretations.^28,40^

The aims of this work are (1) to validate the application of complementary reflectometry techniques in the study of LNP interactions with model endosomal membranes for the first time, (2) to gain new insight into physicochemical processes occurring in the studied system including lipid insertion, LNP binding, and ionizable lipid–nucleic acid complex delivery with respect to the pH and the stage of the endosome model, and (3) to establish a robust physical basis for future work comparing the performance characteristics of a range of newly developed LNP systems with a view to improving the RNA delivery to the cytosol. The work has important implications for the future rational design of LNPs for application as enhanced delivery vehicles of nucleic acids.

## Materials and Methods

### Materials

The lipids 1-hexadecanoyl-2-(9*Z*-octadecenoyl)-*sn*-glycero-3-phosphocholine (POPC; >99% purity), 1,2-distearoyl-*sn*-glycero-3-phosphocholine (DSPC; >99% purity), 1,2-dioleyl-*sn*-glycero-3-phosphoethamolamine (DOPE; 100% purity), *sn*-(3-oleoyl-2-hydroxy)glycerol-1-phospho-*sn*-1′-(3′-oleoyl-2′-hydroxy)glycerol (ammonium salt), also known as bis(monoleoylglycero)phosphate (BMP_18:1_; >99% purity), and sphingomyelin from porcine brain (SM) were all purchased from Avanti Polar Lipids (Alabaster, AL). 1,2-Dimyristoyl-*rac*-glycero-3-methoxypolyethylene glycol-2000 (DMG-PEG-2000) was from NOF America Corp. (New York, NY). Cholesterol (≥99% purity), 2-(*N*-morpholino)ethanesulfonic acid (MES), poly(adenylic acid) [Poly(A); 100–500 kDa], glycerol, phosphate-buffered saline (PBS), and 2-amino-2-(hydroxymethyl)propane-1,3-diol (TRIS) were purchased from Sigma-Aldrich (Poole, U.K.), as were spectroscopic-grade (AnalaR) chloroform and ethanol. Heptatriaconta-6,9,28,31-tetraen-19-yl 4-(dimethylamino)butanoate (DLin-MC3-DMA or MC3) was obtained from Sapala Organics Private Limited (Hyderabad, India), while Firefly luciferase mRNA (FLuc mRNA; 1929 nucleotides, molecular weight = 618.5 kDa calculated assuming an average molecular weight per nucleotide of 320.5 + 159.0) was from CleanCap (TriLink Biotechnologies, San Diego, CA). Ultrapure water was obtained using a Millipore Milli-Q system (Merck Millipore, Darmstadt, Germany) to a resistivity of 18 MΩ cm.

### Preparation of Model Endosomal Monolayers

The compositions of model endosomal monolayers were chosen to mimic endosomal compositions found *in vivo* using, in each case, four lipid components. The EEM comprised 40 mol % POPC, 20 mol % DOPE, 6 mol % SM, and 34 mol % cholesterol, while the LEM comprised 61 mol % POPC, 16 mol % DOPE, 6 mol % BMP_18:1_, and 17 mol % cholesterol.^23,32^ For each type of monolayer, the various lipid components were weighed separately and individually dissolved in chloroform at a concentration of 1 mg mL^–1^. The lipid solutions were then mixed to prepare a spreading solution containing a total of 10 mg of the required lipid mixture. The resulting lipid mixtures were subsequently divided into 1 mg aliquots, dried by evaporating the chloroform under vacuum, and then stored at −20 °C until needed. Immediately prior to every monolayer experiment, a fresh solution of either EEM or LEM lipids was prepared by redissolving the aliquoted lipid in chloroform to a final lipid concentration of 0.1 mg mL^–1^.

### Preparation of LNPs

The LNPs were prepared at room temperature using a Nanoassemblr microfluidic device (Precision NanoSystems, Vancouver, Canada). Stock solutions were first prepared by dissolving the required amounts of DLin-MC3-DMA, DSPC, cholesterol, and DMG-PEG-2000 in ethanol. These stocks were then mixed in a 50:10:38.5:1.5 molar ratio to give a total lipid concentration of 12.5 mM (1.8 mg mL^–1^). FLuc mRNA or Poly(A) was diluted in a 50 mM citrate buffer (pH 3.0) to obtain an mRNA/DNA–lipid weight ratio of 1:20 (cationic ionizable lipid–nucleotide with a 5.7:1 molar ratio). Empty (nucleic acid-free) LNPs were prepared in the same way but using (nucleic acid-free) 50 mM citrate buffer as the aqueous phase. The ethanolic and aqueous solutions were mixed in the Nanoassemblr microfluidic device at a 3:1 volume ratio and a total flow rate of 12 mL min^–1^. The resulting LNPs were dialyzed in PBS overnight at 4 °C, using Slide-A-Lyzer G2 dialysis cassettes (10 kDa molecular weight cutoff; Thermo Scientific, Loughborough, U.K.). Glycerol was added as a cryoprotectant, with the final formulation containing 10% (v/v) glycerol. Samples were aliquoted, frozen, and defrosted prior to the surface pressure and ellipsometry measurements being performed.

### Characterization of the LNPs

The apparent hydrodynamic diameter of the LNPs was determined by dynamic light scattering (DLS) using a Malvern Zetasizer Nano ZS (Malvern Instruments Ltd., Malvern, U.K.). Prior to measurement by DLS, the LNPs were diluted 70-fold using PBS. The resultant apparent hydrodynamic intensity size and polydispersity index (PDI) at 298 ± 0.1 K were recorded at a backscattering angle of 173 °C. The encapsulation efficiency (expressed as %EE) of nucleic acid in the LNPs was quantified by a Quant-iT Ribogreen RNA assay kit (Invitrogen, Paisley, Scotland) following the manufacturer’s instructions. The results of this characterization are shown in Table S1 and discussed under the section entitled Characterization of the LNPs.

### Surface Pressure Measurments

Monolayer experiments were performed at 293 ± 2 K using a G2 Langmuir trough (Kibron, Helsinki, Finland) with a custom-made low-volume stainless steel insert of dimensions 260 × 32 × 1 mm containing a 45 × 32 × 2 mm depression for a tinted glass deflector used to prevent diffuse laser reflections from the trough reaching the detector for the reflectometry measurements. The trough was first cleaned with copious amounts of water, then ethanol, and finally chloroform. It was then filled with 15 mL of an aqueous buffer solution prepared using ultrapure water. The buffers used for different pH values were the following: 5 mM MES buffer for pH 5.5 and 6.5, PBS buffer for pH 7.0 and 7.4, and 10 mM TRIS buffer for pH 8.5. In order to achieve a starting average area per molecule of 120 Å^2^, different amounts (namely, 54 and 64 μL) of 0.1 mg mL^–1^ (total lipid) EEM and LEM mixtures, respectively, were spread using a Hamilton syringe on the surface and left for 15 min to ensure the evaporation of chloroform. With knowledge of the total amount of lipid, and therefore the number of lipid molecules of each type added to the surface, and of the surface area over which the lipid film was spread, it is possible to calculate an average area per molecule (*A*) according to the molar ratio of the various lipid components present. The surface pressure (π) is defined as the difference in the surface tension of a sample compared with that of pure water. The values were measured using a metal alloy Wilhelmy plate and recorded using *Filmware 4.0* software. Furthermore, the reciprocal of the compression modulus (*C*_s_^–1^) is used to evaluate the average phase of the lipid monolayers according to^41^
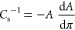
1

It is considered that *C*_s_^–1^ values in the range 12.5–50 mN m^–1^ indicate a monolayer in the liquid expanded phase and values in the range 100–250 mN m^–1^ indicate a monolayer in the liquid condensed phase.^42^

The area over which the lipid was spread was compressed using the barriers at a speed of 120 cm^2^ min^–1^ to a surface pressure of 25 mN m^–1^, where it was held for 2 min before being expanded to 15 m N^1–^ at a speed of 10 cm^2^ min^–1^ and held at constant area for 15 min. This procedure was carried out to ensure that the trough edges and barriers were wetted with lipid, and, hence, the lipid monolayer was stable prior to injections of an aqueous solution of LNPs into the subphase. Once the film was determined to be stable, LNPs were gently injected underneath the lipid monolayer using a syringe with a bent 10 cm needle, and the resulting interaction was constantly monitored for at least 2 h by measuring the variation in the surface pressure. The volume of liquid injected into the subphase in each experiment was 1 mL (an overall volume change of about 6%), namely, a 1 in 16 dilution of the LNPs. All of the experiments were repeated, as indicated in the Supporting Information.

### Ellipsometry

Data were recorded at 293 ± 2 K using an Nanofilm EP4 instrument (Accurion, Goettingen, Germany) with a blue diode laser at a wavelength of 489 nm and an angle of incidence of 50°. Upon reflection of polarized light at the air/water interface, the attenuation (Ψ) and phase shift (Δ) were determined by the optical properties of the system. As opposed to measurements at the solid/water interface where both parameters can be modeled to reveal the interfacial excess and thickness,^39^ only the latter parameter is strongly sensitive to the presence of interfacial material at the air/water interface, the change of which can be related generally to the amount of interfacial material in the thin-film limit of a layer thickness of less than a few tens of nanometers.^35^

The phase shift prior to the injection of LNPs, i.e., for a bare air/water interface or a spread lipid monolayer, Δ_0_, recorded for 10–15 min, was subtracted from the measured values following injection to give the phase shift of the resulting interaction, Δ_int_ = Δ – Δ_0_. This process first compensated for the presence of capillary wave roughness^43^ as well as small systematic errors in the daily calibration of the experiment attributed to instrumental drift and positioning of the deflector and to the starting surface packing of a lipid in the case of interactions with the model monolayers.

Modeling of Δ_int_ to a total amount of interfacial material is highly complex for both multicomponent^44^ and lipid systems:^45^ in the former case, the technique alone cannot distinguish different components, while in the latter case, anisotropy exhibited from lipids present in a condensed phase (or any such lateral domains) strongly influences the data. Further complexities are that lateral heterogeneity in the interfacial material on the micrometer scale, i.e., from different lateral regions of the interface passing in and out of the probed area by the laser (∼1 mm^2^) with time, are exhibited as temporal fluctuations^36^ and that regions of the interface that exceed the thin-film limit can have a negative contribution to Δ_int_ as a result of its periodicity.^46^ As a result of these complexities, in the present work, we retain the phase-shift representation of the data, which we interpret in three contexts. First, the magnitude of Δ_int_ is taken as an approximate measure of the change in the amount of interfacial material per unit area as a result of the interaction. Second, temporal fluctuations above a clear baseline are taken as an indication of thicker regions of the interfacial material or domains of more condensed lipid chains as a result of lipid insertion from LNPs or induced phase separation. Third, temporal fluctuations that are both positive and negative from the baseline are taken as an indication of extended structures in contact with the monolayer as a result of the binding of LNPs. All of the experiments were repeated, as indicated in the Supporting Information.

### BAM

BAM images were recorded at 293 ± 2 K using the same Nanofilm EP4 instrument (Accurion, Goettingen, Germany) with a blue diode laser at a wavelength of 489 nm but in this case at an angle of incidence matching the Brewster angle of the air/water interface of 53.1°. The instrument was used in a mode with p-polarized light, a 10× magnification objective, a polarizer, an analyzer, and a CCD camera. The technique is commonly used to image the lateral dimensions of both anisotropic liquid-condensed domains of phospholipids^47^ and extended structures,^36^ in films at the air/water interface, because the higher reflectivity of both types of features results in lighter features in the resulting images against a darker background. Images were taken at different surface pressures during compression of the EEM and LEM monolayers and at different time points following the injection of LNPs into the subphase while keeping the Langmuir trough barriers stationary. Background was subtracted from the images using an automatic feature of the instrument software with image focusing enabled. The same Γ correction was applied to all of the images to highlight the morphological features of the interfacial material without changing their relative brightness (Irfanview, Germany). It should be noted that BAM has a spatial resolution of several micrometers and, as a consequence, is unable to detect the binding of individual LNPs to the monolayer. All of the experiments were repeated, as indicated in the Supporting Information.

### Statistical Analysis

Statistical analysis of the surface pressure and ellipsometry data was performed using *GraphPad Prism*, version 9. Details of the two-way ANOVA and Šídák’s or Turkey’s multiple comparisons tests are indicated in the Supporting Information. For the surface pressure measurements, the value of the surface pressure at the plateau was averaged across the various repeats and subsequently compared between different experiments (namely, empty vs Poly(A)-loaded LNPs and EEM vs LEM at different subphase pH values). For the ellipsometry measurements, the mean value of Δ_int_ at the plateau for each sample was averaged across repeats and compared across experiments (namely, empty vs Poly(A)-loaded LNPs and EEM vs LEM at different subphase pH values).

## Results

### Predicted Biophysical Processes

**Scheme 1:**
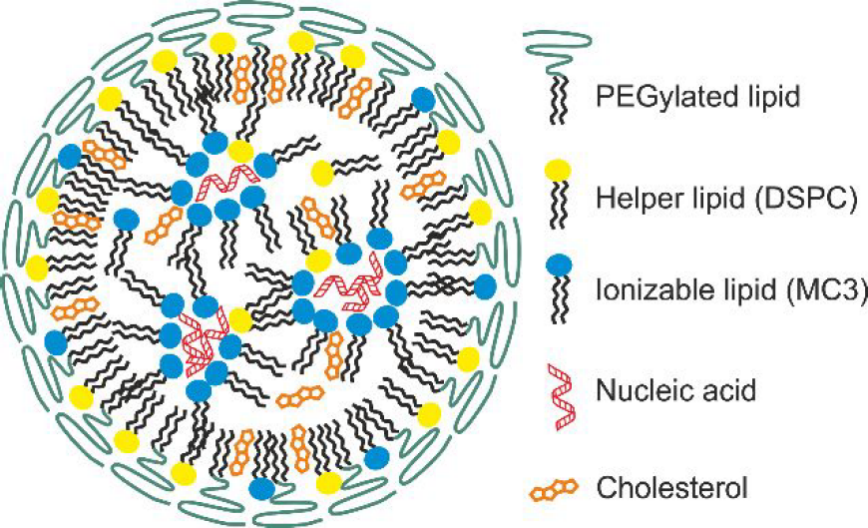
Representation of the Structure and Component Distribution of a LNP

**Scheme 2:**
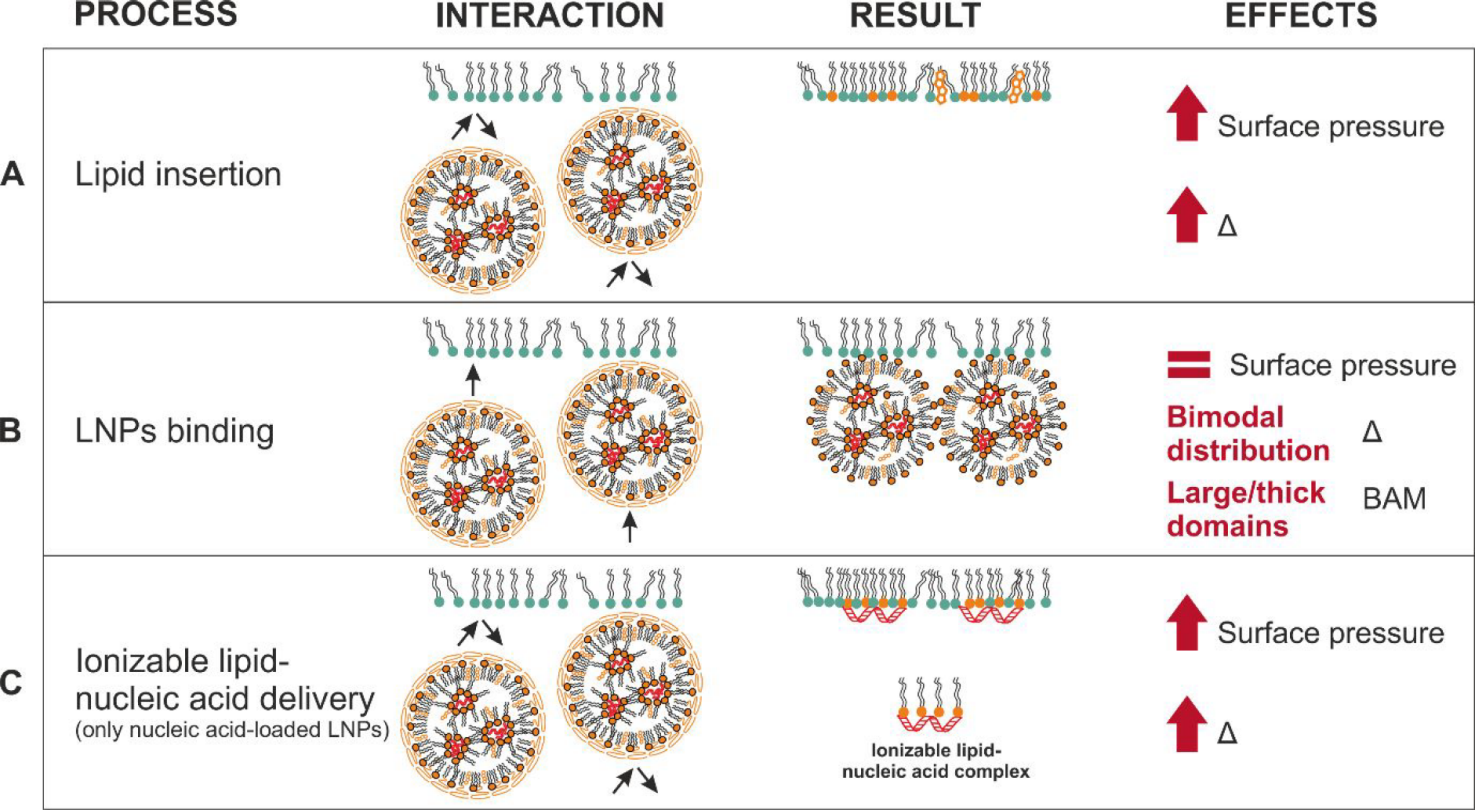
Representation of Three Biophysical Processes, Lipid Insertion (Panel A), LNP Binding (Panel B), and Ionizable Lipid–Nucleic Acid Delivery (Panel C) That Can Occur as a Result of LNP–Monolayer Interactions, Including Sketches of the Molecular Changes during and as a Result of the Interaction, and Stated Effects on the Surface Pressure and Ellipsometry Data Note that the lipids in the model endosomal monolayers have been simplified to a single color (green) and those in the LNPs also to a single color (orange). Panel C can only be applied to nucleic acid-loaded LNPs. LNPs are not drawn to scale.

Before discussing the results, we describe some of the biophysical processes that should be considered for the systems under study and explain how their effects are manifested in the surface pressure and ellipsometry data. With respect to the Langmuir monolayer experiments, an increase in the surface pressure (π) is related to a lowering of the interfacial free energy and, in crude terms, is determined by an increase in the density of the lipid chains in the monolayer.^48^ On the other hand, the phase shift (Δ_int_) obtained from ellipsometry is related to the total amount of interfacial material per unit area, while the nature of the fluctuations in Δ_int_ can reveal additional information, as noted below. Consideration of the possible ways in which LNPs or LNPs’ components (Scheme 1) could interact with the model endosomal membranes led to identification of the three possible processes shown in Scheme 2, namely, lipid insertion, LNP binding, and ionizable lipid–nucleic acid complex delivery.

#### Scheme 2A

**Lipid insertion** occurs when some of the lipid molecules from the LNPs transfer to the monolayer during interactions of the LNPs with the monolayer, which results in an increase in both the lipid chain density in the monolayer and π. The insertion of an additional lipid into the monolayer also results in an increase in Δ_int_ because of the higher total amount of interfacial material per unit area. It should be noted that lipid exchange may also occur, in which the lipid from the monolayer is sequestered by LNPs during the LNP–monolayer interactions; however, the techniques applied in this work are not sensitive to this process.

#### Scheme 2B

**LNP binding** to the lipid monolayer results in an insignificant change in π if the density of the lipid chains in the monolayer is unchanged, although if the binding event is on a time scale that exceeds the measurement time of Δ_int_ of a few seconds, ellipsometry will sense a total interfacial thickness that exceeds the thin-film limit of a few nanometers. The sensitivity of the ellipsometry measurement can be either positive or negative on this length scale because of the periodic wavelength of light, resulting in a bimodal distribution of Δ_int_.

#### Scheme 2C

**Ionizable lipid–nucleic acid complex delivery** from LNPs to the monolayer also results in an increase in both π, because the associated lipid molecules rearrange so that the lipid chains are situated in the surface monolayer, and Δ_int_, because the total amount of interfacial material per unit area increases. However, such a delivery only occurs in experiments involving nucleic acid-containing LNPs and not in those involving nuclei acid-free LNPs or nucleic acid alone.

### Characterization of the Early- and Late-Stage Endosomal Monolayers

**Figure 1:**
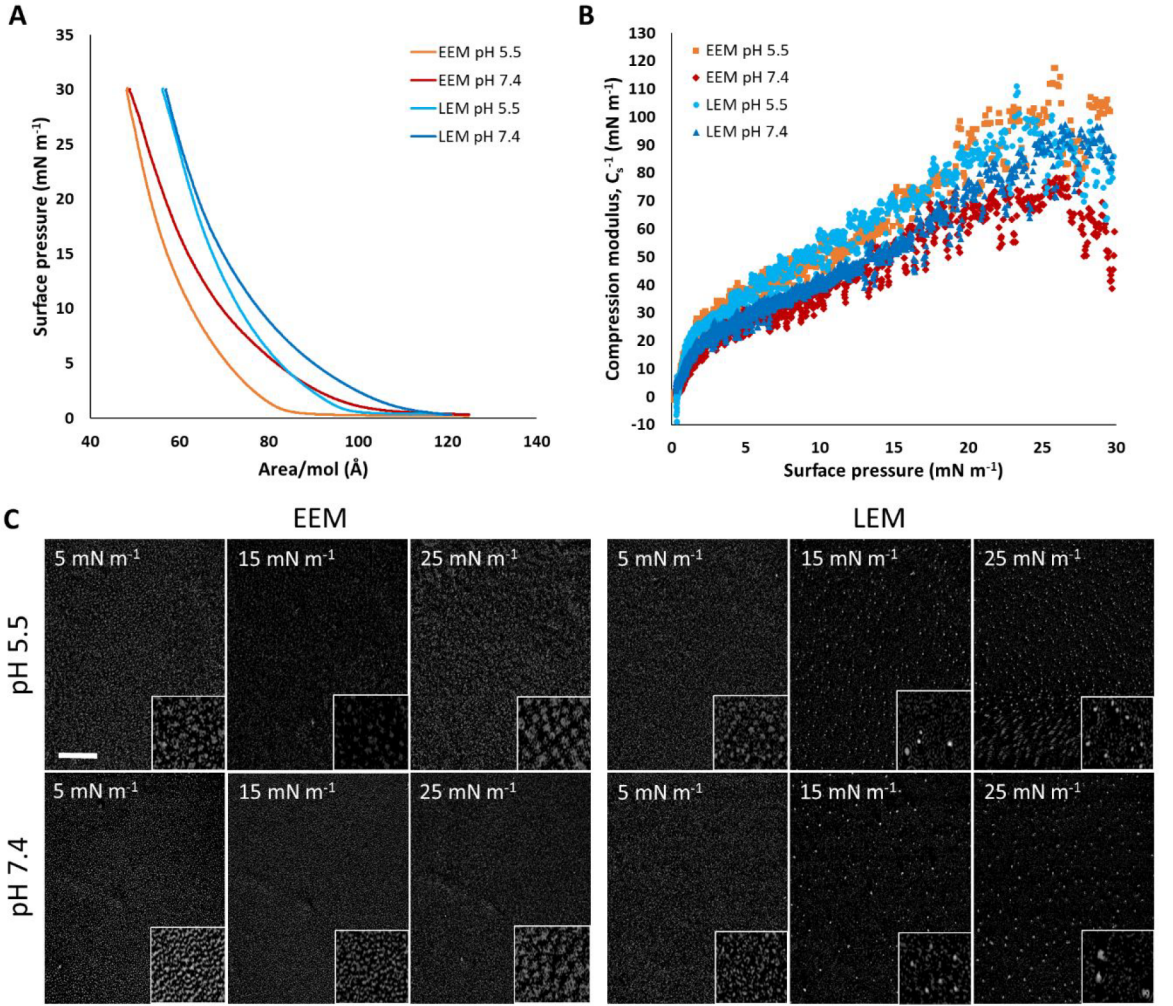
(A) Surface pressure (π)–area (*A*) isotherms for the EEM (orange and red lines) and LEM (light- and dark-blue lines) monolayers on 5 mM MES buffer, pH 5.5 (orange and light-blue lines), and PBS buffer, pH 7.4 (red and dark-blue lines). Note that *A* is the average area for a lipid molecule according to the molar ratio of the lipid components present. (B) Plot of the reciprocal of the compression modulus against surface pressure (EEM of pH 5.5, orange squares; EEM of pH 7.4, red squares; LEM of pH 5.5, light-blue dots; LEM of pH 7.4, dark-blue triangles). (C) BAM images of the EEM and LEM monolayers at varying different surface pressures. The scale bar is 100 μm. The inset images are 3× magnified.

To aid in the interpretation of data from the interaction of LNPs with the EEM and LEM, it was necessary first to characterize the endosomal monolayers in the absence of LNPs at a pH of either 5.5 (the pH of the late endosome) or 7.4 (the pH of the cytosol). The variation in the surface pressure (π) with the average area per molecule (A) of the two endosomal monolayers was determined up to 30 mN m^–1^ (Figure 1A), as was the reciprocal of the compression modulus (Figure 1B). Although the shapes of the isotherms obtained for the two monolayer types are comparable at the same pH, the measured π at the same *A* is larger for the LEM. Regardless of the pH and endosomal type, at π up to 15 mN m^–1^ and with *C*_s_^–1^ of less than 50 mN m^–1^, the shape of the isotherm indicates that the molecules are in a liquid expanded state, which is widely considered to be analogous to the liquid-crystalline state in a lipid bilayer.^49^ As π increases further and *C*_s_^–1^ approaches 100 mN m^–1^, there is a transition of the molecules toward the liquid condensed phase, which is analogous to the gel state in a lipid bilayer.^49^ The differences observed between the two monolayers are attributed to the presence, in the LEM, of 6 mol % anionic BMP_18:1_. BMP_18:1_, a strongly anionic lipid, is only found in the late endosome and lysosome^50^ and will, because of its highly charged nature at both pH values studied coupled with the fact that each of its two alkyl chains contain an unsaturated site, occupy a large area per molecule in the LEM monolayer.

For each monolayer type over the measured range of π, the isotherms obtained at pH 5.5 are more compact in that *A* is generally lower and *C*_s_^–1^ higher at the same π. This effect is likely to be due to the presence of DOPE, because POPC and SM are zwitterionic at both pH values studied, while cholesterol is nonionic in nature and therefore unaffected by the pH. Like BMP_18:1_, DOPE contains two unsaturated alkyl chains and a headgroup whose charge depends upon the pH in that it contains both a phosphate moiety (p*K*_a_ = 1.7) and an amino group (p*K*_a_ ∼ 9.6). Consequently, the amino group becomes increasingly basic as the pH decreases,^51^ while the strongly acidic phosphate group is ionized and therefore negatively charged at all pH values examined here. Consequently, as the pH decreases, the headgroup becomes zwitterionic in nature, occupying a smaller headgroup area.

BAM images of the EEM and LEM monolayers were acquired at both pH values at π values of 5, 15, and 25 mN m^–1^ (Figure 1C). These BAM images serve as a reference for the observed exposure to LNPs. Regardless of the monolayer type or pH, bright spots of a few micrometers in size became increasingly abundant with increasing π. The presence of these domains in the EEM are consistent with domains recorded for lipid mixtures of similar composition.^52^ The EEM contains cholesterol (34 mol %), which is known to have a condensing effect in plasma membranes (and regulate lipid segregation), as does SM (6 mol %). Indeed, domains formed in a four-component monolayer of composition similar to that of the EEM were considered to be a result of the mixing of SM (a high chain-melting lipid) with cholesterol and the demixing of POPC (a low chain-melting lipid) with cholesterol.^53^ On the plasma membrane of animal cells, SM is known to form microdomains with cholesterol and other glycosphingolipids, yielding so-called lipid rafts.^54^ The domains of the LEM, on the other hand, look different in that at surface pressures above 5 mN m^–1^ very small and bright spots appear and subsequently remain at all of the higher pressures tested. These domains are smaller, yet more condensed, compared to those in the EEM. Together, these observations are consistent with the values of *C*_s_^–1^ and lack of a pronounced phase transition in the monolayers under study.

### LNPs Interacting with the Air/Water Interface as a Function of the pH in the Absence of a Model Endosomal Monolayer

**Figure 2:**
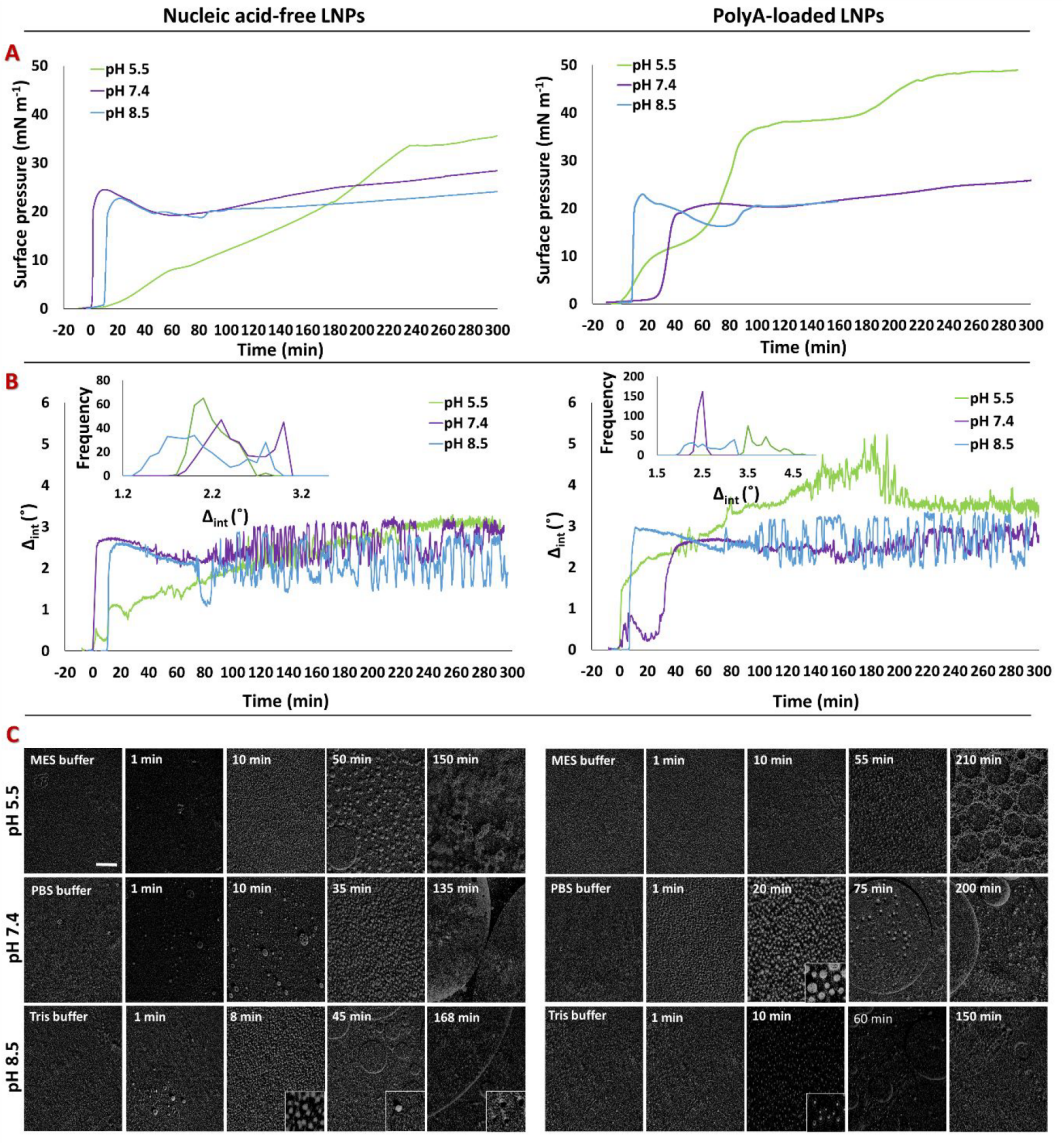
Changes in the (A) surface pressure and (B) Δ_int_ and (C) BAM images of nucleic acid-free LNPs (left panels) and Poly(A)-loaded LNPs (right panels) at a final RNA concentration equivalent to 1 μg mL^–1^ monitored over time at pH 5.5 (5 mM MES; light-green lines), 7.4 (PBS; purple lines) and 8.5 (PBS; light-blue lines). Injection was performed at time 0. The insets in part B are the frequency distribution of each Δ_int_ value recorded between 60 and 150 min. The BAM images were acquired at the stated time after injection of the LNPs. The scale bar is 100 μm. The insets are 3× magnified.

The interaction of LNPs [with Poly(A)] with a bare air/water interface at pH values of 5.5, 7.4, and 8.5 was examined for up to 5 h after injection into the subphase (Figure 2) by monitoring π (Figure 2A) and Δ_int_ (Figure 2B) and taking BAM images (Figure 2C). LNPs were also prepared with no nucleic acid cargo to help understand the role of the nucleic acid cargo in the interaction with model endosomal monolayers. It is worth noting that, while the surface pressure reached at the plateau is extremely consistent between replicates, there was more variability in the lag phase, i.e., time to liftoff as well as the rate of increase in surface pressure, and as a consequence, therefore only trends are reported here.

Most noticeably, at the lowest pH of 5.5, the addition of both nucleic acid-containing and nucleic acid-free LNPs resulted in large increases in Δ_int_ and π, although the maximum changes in the value were only reached 4 h after LNP injection. In contrast, at the higher pH values of 7.4 and 8.5, the changes in Δ_int_ and π recorded were smaller but quicker, with maximum values being reached within 40 min of LNP injection (Statistical Analysis and Figures S7 and S8). At pH 7.4, the fluctuations in Δ_int_ after interaction, at the air/water interface, with nucleic acid-free LNPs were greater than those after interaction with Poly(A)-loaded LNPs, while both interfaces exhibit comparable fluctuations at pH 8.5. These fluctuations start about 80 min after LNP injection, with Δ_int_ values varying about a mean, as demonstrated by the bimodal distribution of the values (inset in Figure 2B). Such a bimodal distribution of the ellipsometry values indicates that, over time, the interfacial material adopts a morphology with regions that have considerably different thicknesses within the plane of the interface. This is the case of laterally segregated domains, which move in and out of the laser beam with time, possibly as a result of Marangoni flow from spreading events.^55^ The distinct values of the bimodal distribution suggest that, over the first hour of the interaction, the lateral size of the domains increases to the millimeter length scale, matching the size of the interfacial area probed by the laser.

Together these observations suggest that, after LNP injection, the lipids comprising LNP transfer to the air/water interface cause increases in π and Δ_int_. This transfer is clearly influenced by the pH, occurring more slowly, but to a greater extent, at pH 5.5. At this pH, Poly(A)-loaded LNPs resulted in higher values of π and Δ_int_ compared to nucleic acid-free LNPs, while no such difference was observed at the higher pH values of 7.4 and 8.5. At a pH of 5.5, the MC3 lipid becomes predominately ionized (cationic), possibly causing the remaining LNPs to become unstable,^20^ releasing their component lipids and, when present, forming water-insoluble complexes of MC3 and nucleic acid.^7^

At pH 7.4 and 8.5, although the PEGylated lipids will be shed upon injection, MC3 is predominately un-ionized. Therefore, less disintegration of the LNPs is expected at these pH values and, when Poly(A) is present, less formation and lower delivery of ionizable lipid–nucleic acid complexes. This hypothesis can explain the lower increase in π and the nature of the fluctuations observed in Δ_int_ and the similarity in behavior of both the Poly(A)-loaded and nucleic acid-free LNPs at these pH values.^7^

This hypothesis is further supported by the BAM experiments, where images taken at the late time points at pH 7.4 and 8.5 showed the presence of much larger domains than those seen at pH 5.5, indicating that the reduced LNP disintegration at higher pH values results in the formation of large thick domains at the air/water interface. Regardless of the pH and the absence/presence of Poly(A), a few minutes after injection of the LNPs, the BAM images show the appearance of domains of a few microns in size, which considerably increase in size over the next couple of hours. The one exception to this is the Poly(A)-loaded LNPs at pH 5.5 at 210 min after injection, where a distinct extended network structure is observed. Such a feature is similar to those seen in mixed films containing macromolecules,^56,57^ and we infer that the domains may be formed due to the presence of water-insoluble ionizable lipid–nucleic acid complexes at the air/water interface.

In summary, the following observations can be made:

(i) Higher π and Δ_int_ were reached at pH 5.5 compared to pH 7.4 and 8.5, as a result of MC3 protonation, LNP disruption, and lipid insertion and ionizable lipid–nucleic acid delivery [Poly(A)-loaded LNPs only] to the surface.

(ii) Poly(A)-loaded LNPs reached higher π and Δ_int_ compared to nucleic acid-free LNPs but only at pH 5.5, thereby demonstrating the impact of the lipid–nucleic acid complex formation and delivery.

(iii) The fluctuations in Δ_int_ recorded at pH 7.4 and 8.5 are the result of domain formation, as observed with BAM, due to whole LNP translocation to the surface.

### Nucleic Acid-Free LNPs Interacting Similarly with Monolayer Models of the Early and Late Endosomes

**Figure 3:**
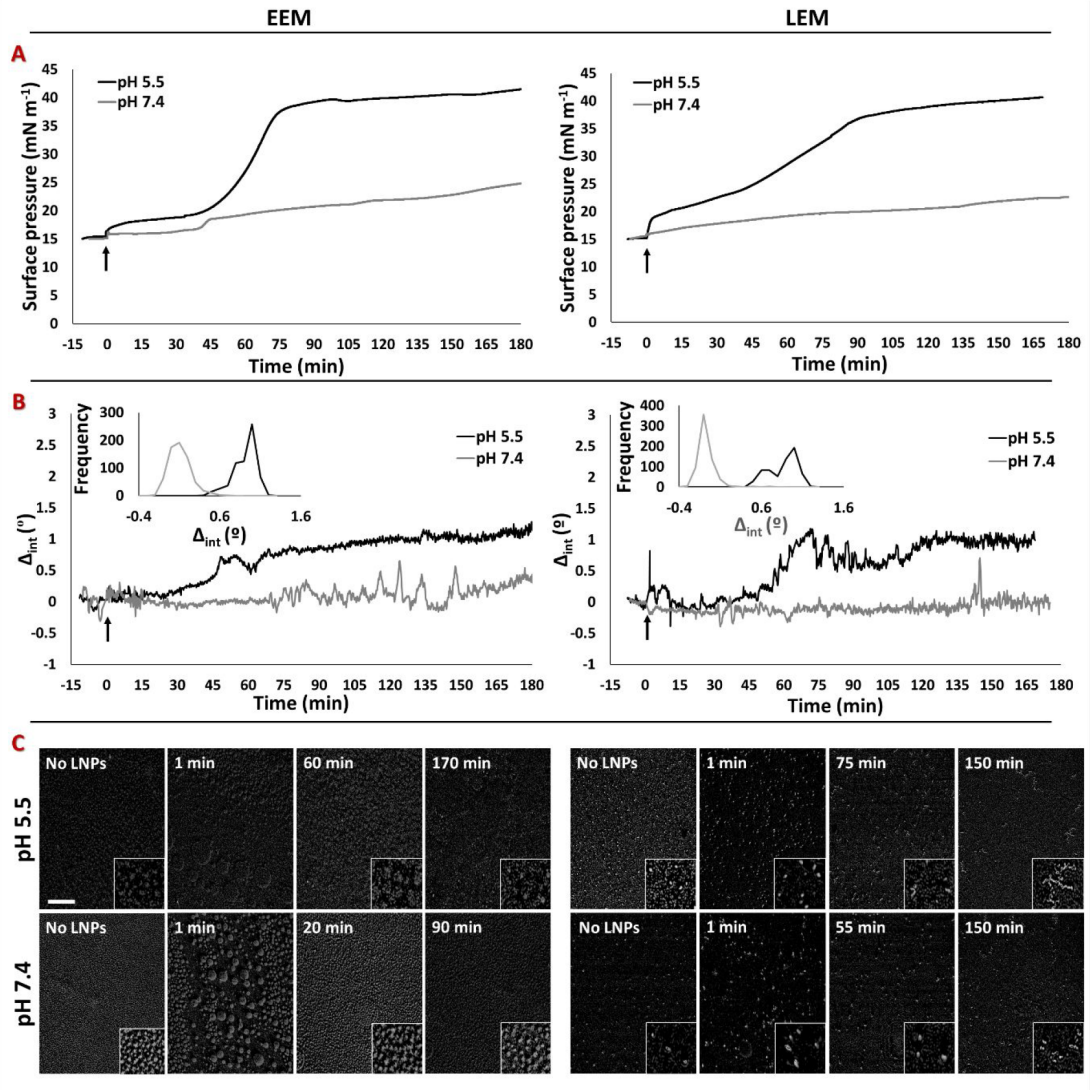
Changes in the (A) surface pressure and (B) Δ_int_ and (C) BAM images of models of the EEM (left panels) and LEM (right panels) monolayers at pH 5.5 (5 mM MES buffer) after the injection of nucleic acid-free LNPs at a concentration equivalent to that of LNPs containing 1 μg mL^–1^ RNA. Injection was performed at time 0 (black arrows indicate injection). The insets in the middle panels are the frequencies of Δ_int_ between 60 and 180 min. The BAM images were acquired at the stated time after injection of the LNPs. The scale bar is 100 μm. The insets are 3× magnified.

The interaction of nucleic acid-free LNPs with the two model endosomal monolayers was examined after their injection into a subphase at either pH 5.5 or 7.4 (Figure 3). A starting π of 15 mN m^–1^ was selected as a compromise between a higher, more biologically relevant π of around 30–35 mN m^–1^ ^58^ and a lower value that results in a more significant change in π.^59,60^ In general, π was monitored for at least 2 h, and in some cases up to 5 h, after injection. It should be noted that the injection of a pure buffer under the EEM and LEM at pH 5.5 was investigated as a control to ensure that the injection caused no mechanical disturbance to the monolayer and introduced no artifacts into the data (Figure S1).

In the presence of model endosomal membranes at pH 5.5, a lag phase was observed before any interaction of the nucleic acid-free LNPs was seen, and although this interaction was slow, it was strong (Figure 3A). In the case of the EEM, a lag of about 45 min was seen before π and Δ_int_ increased, whereupon the interaction continued for a total of 75 min until maximum values of Δ_int_ and π (40 mN m^–1^) were recorded. With the LEM, comparable maximum values of Δ_int_ and π (38 mN m^–1^) were observed about 90 min after LNP injection.

When LNPs were injected under an EEM at a subphase of pH 7.4, a lag of about 45 min was seen before any change in π occurred, whereupon π increased to 18 mN m^–1^, further increasing to 25 mN m^–1^ after 3 h. Δ_int_ showed a slight increase but only at later time points, at which time some temporal fluctuations were observed (Figure 3B). By way of comparison, the lag phase and fluctuations in Δ_int_ seen for a LEM were minimal, with π increasing very slowly up to about 20 mN m^–1^ at 3 h after LNP injection, with only a very small change in Δ_int_ being observed. These observations suggest that at pH 5.5 material from LNPs was incorporated into the model membranes, but only over an extended time period. This time lag was comparable to the data obtained for the interaction of nucleic acid-free LNPs at the bare air/water interface at pH 5.5 (Figure 2B) when a π of 15 mN m^–1^ was reached 2 h after LNP injection, with a maximum value of around 35 mN m^–1^ being observed at 4 h.

In contrast, at pH 7.4, the Δ_int_ fluctuations observed with the EEM suggest the formation of domains as seen for the LNPs alone. Significantly, at both pH values, the interaction of nucleic acid-free LNPs with either the EEM or LEM monolayers is qualitatively similar to no obvious differences being recorded for the two membrane types.

The BAM images (Figure 3C) highlight that, prior to LNP injection at pH 5.5, micrometer-sized domains were present in the monolayers However, upon injection, there was a rapid formation of larger domains, which remained only for a few minutes, providing further evidence for the proposed lipid exchange. When a plateau in π of about 40 mN m^–1^ was reached, the domains were less visible, probably as result of the insertion of lipids from the LNPs into the monolayer. Interestingly, this phenomenon was not observed at the higher pH of 7.4 because the domains remained at the later time points, with some domains appearing occasionally in the field of view of the camera (Figure S2); these domains are attributed to the fluctuations in Δ_int_ observed for the EEM. For the LEM at both pH values, bright spots are the most evident feature even after LNP injection, although they tend to become more sparse over time as π increased.

In summary, the following observations can be made:

(i) Nucleic acid-free LNPs injected underneath the EEM or LEM at pH 5.5 resulted in much higher π and Δ_int_ values compared to the corresponding system at pH 7.4 as a result of MC3 protonation, LNP disruption, and lipid components from LNP insertion into the monolayer (Scheme 2A).

(ii) At pH 7.4, the injection of nucleic acid-free LNPs below the EEM showed some Δ_int_ fluctuations, indicating the formation of domains, as confirmed by the BAM data, due to whole LNP binding (Scheme 2B).

### Subphase pH Affecting the Extent of Interaction of Poly(A)-Loaded LNPs with Monolayers of Endosomal Membranes

A range of subphase pH values (5.5, 6.5, 7.0, 7.4, and 8.5) were selected to determine in more detail the effect of the pH on the interaction of LNPs with the two model endosomal membranes. Because different buffers were used to obtain these different pH values, it was necessary to ensure the absence of any effects due to the varying nature of the buffer. As a control measurement, therefore, a comparison was made of the results obtained using 5 mM MES and PBS buffers at pH 5.5 (Figure S3) to exclude any buffer related effects. Because the effects of LNPs on the behavior of the EEM and LEM were similar, both are discussed together.

**Figure 4:**
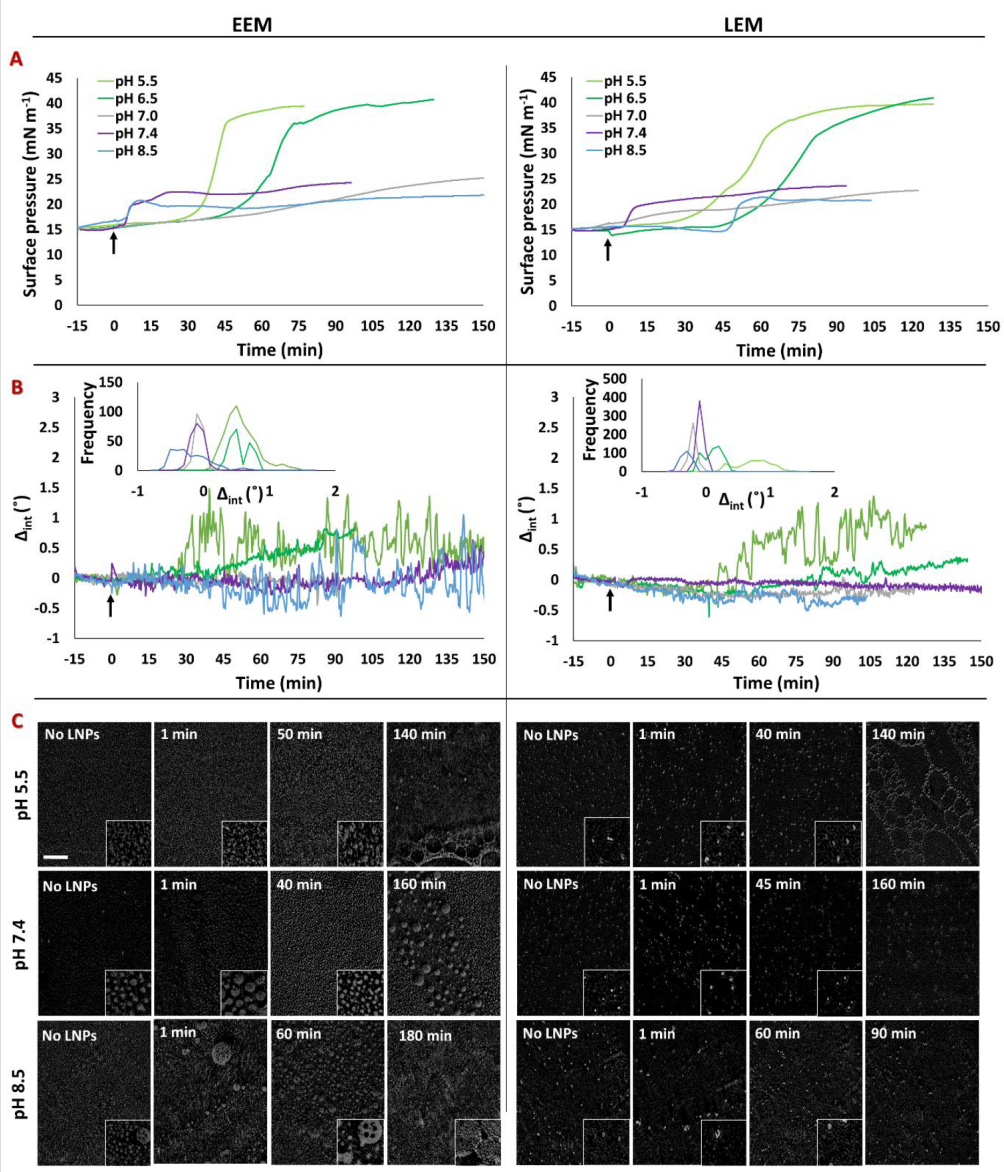
Changes in the (A) surface pressure and (B) Δ_int_ and (C) BAM images of the EEM (left panels) and LEM (right panels) at pH 5.5 (5 mM MES buffer, light-green line), 6.5 (5 mM MES buffer, dark-green line), 7.0 (PBS buffer, gray line), 7.4 (PBS buffer, purple line), and 8.5 (10 mM TRIS buffer, light-blue line) after injection of Poly(A)-loaded LNPs to a final RNA concentration of 1 μg mL^–1^. Injection was performed at time 0 (black arrows indicate injection). The insets in the middle panels are the frequencies of Δ_int_ from 60 min until the end point. BAM images were acquired at the stated time after injection of the LNPs. The scale bar is 100 μm. The insets are 3× magnified.

At pH 7.0 and 7.4, the changes in π of both endosomal membranes after injection of the LNPs were modest (Figure 4A), equilibrating at a value of just 22 mN m^–1^, suggesting only a limited insertion of LNP lipids into the monolayers. Similarly, the temporal fluctuations in Δ_int_ were minimal, indicating either the absence or limited phase separation of the lipid and the formation of limited size domains (Figure 4B).

At pH 8.5, the data are subtly different. Specifically, while a modest change in π indicates a limited insertion of LNP lipids into each of the monolayers, the positive and negative temporal fluctuations of Δ_int_ observed for the EEM indicate that the interfacial film contains material that possesses a thickness greater than the thin-film limit of a few tens of nanometers. This finding is in line with the situation observed with LNPs in the absence of an endosomal monolayer at pH 8.5 (Figure 2B).

At pH 5.5 and 6.5, the observed differences are even more pronounced. Here, after a lag phase, there is a sharp increase in π to around 40 mN m^–1^, which is mirrored by an increase in the baseline value of Δ_int_ and which is consistent with an increase in the lipid density per unit area (Statistical Analysis and Figures S5 and S6).

BAM images of the interactions were acquired at three pH values, representative of the different types of behavior discussed above (Figure 4C). The images highlighted that the characteristic small domains of the EEM remained visible, even after LNP injection. At pH 7.4, significant changes over time were not observed, consistent with the minimal effects of interaction inferred above. However, at pH 8.5, large domains were observed at later time points, confirming the phase separation inferred from ellipsometry. The very bright spots typical of LEM monolayers were also observed after LNP injection at every pH value measured. The distinct laterally extended network structures seen for Poly(A)-loaded LNPs at the bare air/water interface (Figure 2C) were also seen for both types of endosomal membranes at pH 5.5 after an increase in π due to the presence of Poly(A)-loaded LNPs, which plateaued at about 140 min (Figure 4C).

In summary, the following observations can be made:

(i) At pH <7, significant increases in π and in the baseline of Δ_int_ were recorded after the injection of Poly(A)-loaded LNPs underneath the EEM and LEM monolayers as a result of MC3 protonation, LNP disruption, lipid insertion, and ionizable lipid–nucleic acid delivery (Scheme 2A,C)

(ii) At pH 5.5, pronounced fluctuations in Δ_int_ were observed, consistent with the formation of laterally extended network structures at late time points, as observed with BAM.

(iii) At pH >6.5, only minimal changes in π and Δ_int_ were recorded, except at pH 8.5 with the EEM, where fluctuations in Δ_int_ and larger domains with BAM were observed.

### Nucleic Acid Cargo of the LNPs Affecting Their Extent of Interaction with the Model Endosomal Membrane at pH 5.5

**Figure 5:**
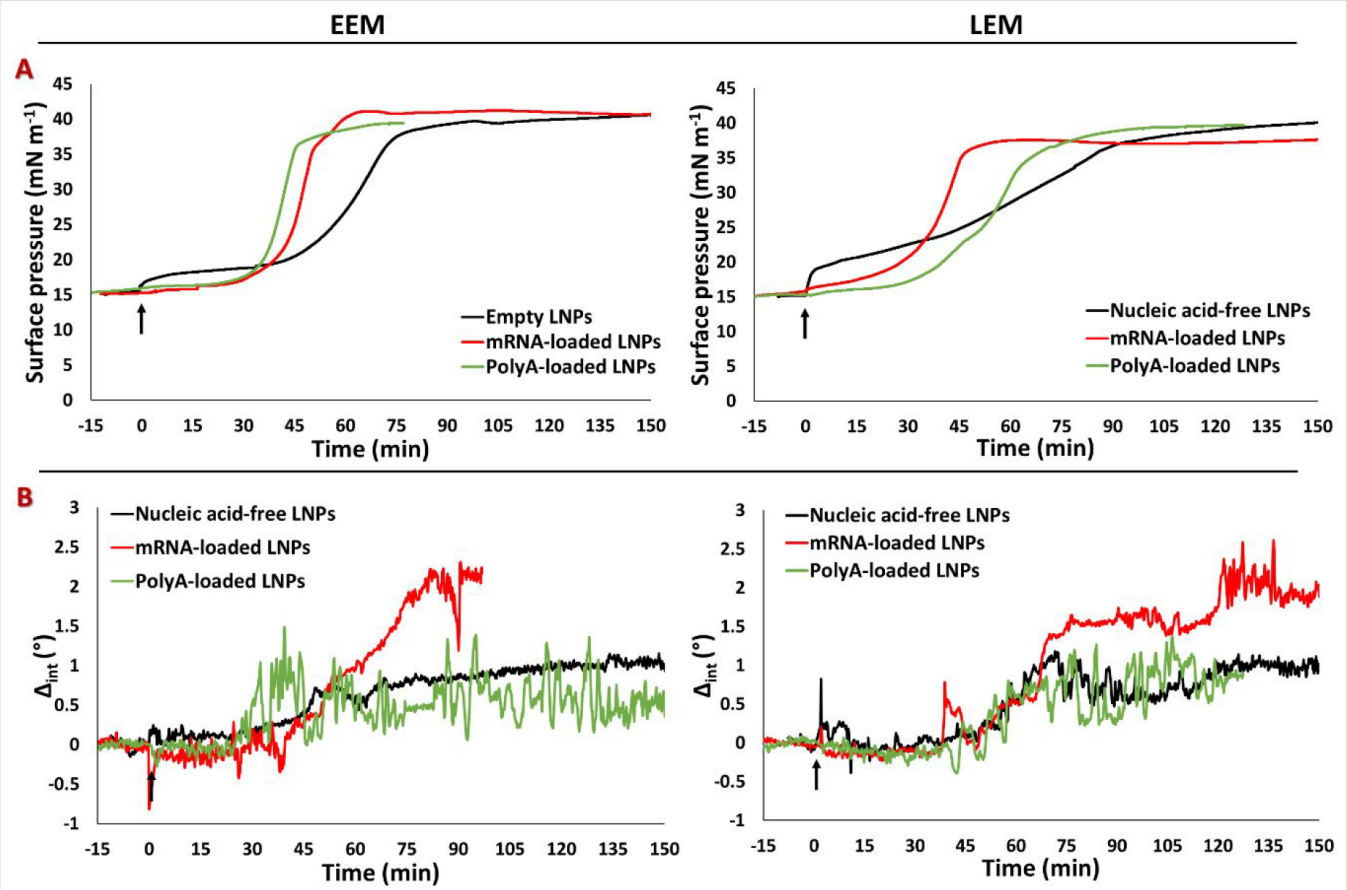
Changes in the (A) surface pressure and (B) Δ_int_ of the EEM (left panels) and LEM (right panels) at pH 5.5 (5 mM MES buffer) after the injection of nucleic acid-free, mRNA-loaded, or Poly(A)-loaded LNPs at a final concentration of RNA of 1 μg mL^–1^. Injection was performed at time 0 (black arrows indicate injection).

Figure 5A gives a comparison of the changes in π and Δ_int_ of mRNA-loaded LNPs and Poly(A)-loaded and nucleic acid-free LNPs at pH 5.5. The interaction of mRNA-loaded LNPs with the two model monolayers was comparable to that of the Poly(A)-loaded LNPs in terms of π. In fact, their π values increased more quickly compared to those of the nucleic acid-free LNPs, with lag phase times of 30 min for EEM and 30–40 min for LEM, reaching 40 and 35 mN m^–1^, respectively, about 1 h after LNP injection. Furthermore, the duration of the lag phase observed with ellipsometry (Figure 5B) was comparable. As observed above, Poly(A)-loaded LNPs showed pronounced fluctuations in Δ_int_, and, interestingly, the baseline of Δ_int_ of mRNA-loaded LNPs exhibited a greater increase than the Poly(A)-loaded and nucleic acid-free LNPs. This is interpreted as a greater extent of delivery of the MC3 lipid–nucleic acid complex, implying that the effect of mRNA on the interface is greater, meriting further work on the subject. On the other hand, no significant differences were observed between the three types of LNPs with either endosomal membrane at pH 7.4 (Figure S4), thus proving the crucial role played by the pH-sensitive ionizable lipid MC3 in the interaction with model endosomal monolayers.

In summary, the following observation can be made:

(i) Although the same π was reached, higher Δ_int_ values were exhibited at pH 5.5 with mRNA-loaded LNPs compared to nucleic acid-free and Poly(A)-loaded LNPs, indicating that larger nucleic acid molecules may be able to interact more strongly with ionizable lipid molecules and, consequently, interact more with model endosomal membranes.

## Discussion

While the importance of endosomal escape for effective intracellular delivery has been unequivocally established, little is known about the mechanism of interaction of the LNPs with endosomal membranes, assumed to be a prerequisite for endosomal escape. To our knowledge, no one has reproduced the interaction of LNPs with model endosomal membranes as we have done here or has elucidated the physicochemical processes that take place with respect to the pH, the stage of the endosome, and the nature of the nucleic acid cargo. To do this, we have used a combination of a Langmuir trough using ellipsometry and BAM to gain new information on processes including LNP binding, lipid insertion, and nucleic acid delivery. Here, we used LNPs of the same lipid composition as those examined previously by others^20,21^ to allow us to relate the structure of the LNPs to their interaction with the model endosomal membranes.

The effects of the subphase pH are dramatic. We have shown that, on the basis of π and Δ_int_, the strongest interactions occur between LNPs (with/without a nucleic acid payload) at less than or equal to pH 6.5 for both the EEM and LEM. A key feature of the data, however, is an extended lag phase.

The origin of the lag phase observed at acidic pH is unclear. Therefore, we propose two possible reasons for this. In the first, PEGylated lipids are desorbed from the LNP surface while any MC3 is ionized (positively charged), possibly inducing instability in the remaining LNPs.^20^ This would allow the LNP components to diffuse into the air/water interface and interact with the model endosomal monolayers. In this context, it has been previously reported that LNPs of similar composition took about 15 min to shed about 50% of their PEGylated lipid molecules *ex vivo*.^61^ In the second, exchange of lipid material occurs between the LNPs and the endosomal monolayers, as was observed in a study of the interaction of cubic phase nanoparticles with a solid-supported lipid bilayer.^62^ The result would be no net change in π or Δ_int_ until sufficient lipid exchange has occurred, whereupon the packing density of the monolayer increased, indicating that favorable lipid insertion occurs.

It was mentioned that, in acidic conditions, MC3 is present in its cationic form, which, together with PEG shedding, could result in disintegration of the LNPs. It has been shown that MC3, when positively charged, can form a water-insoluble complex with nucleic acid,^7^ which would then be available to interact with anionic lipids present in the model endosomal membrane. The injection of free Poly(A), i.e., unentrapped in an LNP, gave results comparable to those of the control buffer injection, proving that nucleic acid on its own does not have any influence on the observed increase in π and Δ_int_ (Figure S1). Therefore, MC3 lipid–nucleic acid complex delivery plus insertion of other components of the LNPs are attributed to the pronounced increase in π and the lipid density per unit area observed at pH values equal or below 6.5 (Figure 4A,B).^63^

At pH >7.0, the interactions appear to be suppressed because lower values of π and Δ_int_ values are reached, with thick domains of material observed at extended interaction times with BAM and fluctuations in Δ_int_ observed at pH 8.5 and, in some cases, at pH 7.4, in either the presence or absence of endosomal membranes (Figures 2B, 3B, and 4B). These results together indicate that MC3 is the key component in LNP fusion with the EEM and LEM at lower pH, a step that is crucial to triggering endosomal escape of its nucleic acid cargo.^64^ At higher pH, the LNPs can remain intact at the interface (Scheme 2). It still remains to be clarified, however, how the pH affects the PEG stability/desorption, particularly because it was previously seen that buffer ions have an effect on PEG–lipid interactions with lipid bilayers.^65^

Perhaps surprisingly, the effect of the stage of the endosomal membrane is minimal. Although in some cases there were small differences in the extent of interaction between the two model membranes, where in such cases, the interaction of the LNPs with the EEM was favored. For example, at pH 6.5, the extent of interaction of Poly(A)-loaded LNPs with the EEM was slightly greater than that seen with the LEM (Figure 4B). This is a significant finding because pH 6.5 is typical of the early endosomes, while lower pH values are found in the late endosomes; coupled with this is the fact that it has been demonstrated that LNPs need to escape at an early stage of the endosomal pathway in order to prevent LNP accumulation, which was shown to suppress endosome acidification, therefore reducing escape and inducing cytotoxicity.^66^

Last, the effects of the nature of the LNP cargo are also revealing. Our data show that mRNA-loaded LNPs exhibit a stronger interaction than Poly(A)-loaded LNPs with the model endosomal membranes at pH 5.5. In this case, Δ_int_ reaches higher values, while π is unchanged, which together imply the formation of a more substantial (i.e., thicker) layer, attributable to a greater amount of nucleic acid bound to the cationic lipid head groups delivered to the surface. Arteta et al. showed that mRNA is located inside water cylinders in the core of the LNPs, interacting electrostatically with the surrounding MC3 lipid;^21^ indeed, nucleic acid-free LNPs were reported to have a more ordered inverse droplet micellar core, while RNA-loaded LNPs have an inverse wormlike micellar core.^21^ This observation was recently confirmed by Sebastiani et al.^22^ Because the FLuc mRNA used here has a molecular weight of 618.5 kDa, larger than the Poly(A) used at 100–500 kDa, it is assumed that each mRNA molecule will interact with more MC3 molecules than Poly(A) will. As a consequence, upon interaction with the endosomal monolayer, a more substantial layer forms at the air/water interface. In fact, it was previously reported that only weak electrostatic interactions are exhibited between nucleic acid and MC3 for siRNA-loaded LNPs compared to LNPs loaded with mRNA and Poly(A).^67^

## Conclusions

In the present work, we have investigated the interactions of LNPs that contain ionizable lipids with model endosomal membranes using the platform of a Langmuir trough as a basis for the application of reflectometry techniques for the first time. Physicochemical information on the interactions was resolved through a comparison of the surface pressure with the optical phase shift from ellipsometry and information on the interfacial morphology provided by BAM imaging. The approach has allowed us to understand the relative importance of the effects of the subphase pH, the stage of the endosome, and the nucleic acid cargo, which were until now unknown. The combination of techniques applied in the present work has allowed us to elucidate LNP binding, lipid insertion, and nucleic acid delivery mechanisms of the system. Although clearly outside the scope of the present work, it may be noted that, through the use of selective deuteration and careful experimental design, a technique like neutron reflectometry could also be applied to such systems to provide information on which lipids are exchanged between the LNPs and model endosomal membranes during the course of the interaction.^62^ While the present results clearly support the importance of the endosomal pH in the effectiveness of LNPs in deliverying their nucleic acid cargo, the influence that the nature of the nucleic acid had on its delivery was unexpected. Our data imply that the extent of delivery of mRNA to the model membranes is greater than that of Poly(A), a result that can be rationalized in terms of the charge density of nucleic acid.^68^ To our knowledge, this observation has not been previously reported. A factor that surprisingly showed a relatively small effect on the resulting interactions is the stage of the endosome modeled. Although some small differences were observed, it was generally shown that the nature of the interactions are broadly equivalent in the early- and late-stage endosomes. Taken together, these observations imply that, in order to further improve the delivery of nucleic acid, there is a need to develop delivery systems that more rapidly release their cargo at the endosomal pH to increase the possibility of endosomal release and to optimize their design to match the nucleic acid it is carrying.

## Supporting Information Available

Characterization of LNPs, control Langmuir experiments, and statistical analysis. (PDF
