# Supplementary material for: Nucleic Acid-Loaded Lipid Nanoparticle Interactions with Model Endosomal Membranes
Source: ACS Appl Mater Interfaces. 2022 Jun 27;14(26):30371–84. doi: 10.1021/acsami.2c06065 (PMC9264317; doi:10.1021/acsami.2c06065)
Supplement: Supplementary file 1 — am2c06065_si_001.pdf [file am2c06065_si_001.pdf]

SUPPORTING INFORMATION

Nucleic acid-loaded lipid nanoparticle interactions with model endosomal membranes

Alice Spadea[a,b], Mark Jackman[c], Lili Cui[c,], Sara Pereira[c],

M. Jayne Lawrence[a,b,*], Richard A. Campbell[b,*] and Marianne Ashford[d]

[a]NorthWest Centre for Advanced Drug Delivery (NoWCADD), School of Health Sciences, University of Manchester, Oxford Road, Manchester, UK,

[b]Division of Pharmacy and Optometry, Faculty of Biology, Medicine and Health, University of Manchester, Oxford Road, Manchester, UK,

[d]Advanced Drug Delivery, Pharmaceutical Sciences, R&D, AstraZeneca, Cambridge, UK,

and

[c]Advanced Drug Delivery, Pharmaceutical Sciences, R & D, AstraZeneca, Macclesfield, UK.

*Corresponding authors: Jayne Lawrence, jayne.lawrence@manchester.ac.uk

Richard Campbell, richard.campbell@manchester.ac.uk

Index **Page**

Characterisation of the LNPs	3
Control Langmuir Experiments	4
Statistical Analysis	7
References	9

Characterisation of the LNPs

The apparent hydrodynamic size of the various types of LNPs prepared, which were very reproducible between batches, clearly vary according to whether any nucleic acid was present and, if so, the nature of the nucleic acid (Table S1). The observation that the nucleic acid-containing LNPs were significantly larger than nucleic acid-free ones shows that the cargo affects the volume of the particles, possibly influencing the LNPs internal structure.[1] Surprisingly, the largest sized nucleic acid, mRNA FLuc (Poly(A) 100-500 kDa; FLuc 1929 nucleotides, 618.5 kDa) produced smaller sized LNPs than those prepared using the smaller and more polydisperse nucleic acid, Poly(A). Previously, it has been reported that LNPs formulated with larger molecular weight mRNA were found to be larger than the values reported for LNPs containing lower molecular weight siRNA.[2, 3] The results of the present study suggest that the larger size of the Poly(A)-loaded LNPs may be a consequence of the differing persistance lengths of the two nucleic acids as Poly(A) is known to exhibit pH dependent behaviour transitioning from a single to double stranded helical structure upon moving into an acidic environment.[4] Very low polydispersities (expressed as a polydispersity index, PDI) were observed for the nucleic acid containing LNPs in contrast to the higher values seen with their corresponding nucleic acid-free counterparts. The smaller size and much higher polydispersity of the nucleic acid-free LNPs compared to FLuc containing LNPs of the same composition have been previously reported.[5] Regardless of whether Poly(A) or mRNA was loaded into the LNPs, the resultant encapsulation efficiency (EE%) was in the range 95-99%, consistent with previous studies.[6]

Table S1: Physicochemical properties of the LNPs (lipid:nucleic acid weight ratio 20:1) used in the study.

**Nucleic acid**	**Apparent hydrodynamic size (nm)**	**Polydispersity Index (PDI)**
None	53 ± 1.5	0.19 ± 0.03
None	52 ± 1.3	0.13 ± 0.03
Poly(A)	81 ± 0.1	0.06 ± 0.09
Poly(A)	83 ± 0.7	0.05 ± 0.01
Poly(A)	77 ± 0.5	0.05 ± 0.03
FLuc	62 ± 1.9	0.06 ± 0.02
FLuc	63 ± 1.4	0.1 ± 0.02
FLuc	70 ± 1.5	0.09 ± 0.03

Control Langmuir Experiments

The injection procedure was observed to cause minimal temporary changes in surface pressure of 2 mN m^{-1} (black lines; Figure S1 top panels) and no observable temporary changes in Δ_{int} (black lines; Figure S1 bottom panels).

As a reference measurement, the injection of free Poly(A) (final concentration 1 µg ml^{-1}) was investigated for the same monolayers at pH 5.5. The increase in surface pressure compared with the control measurement is about 2 mN m^{-1} for the EEM and about 4 mN m^{-1} for the LEM. Additionally, although the mean value of Δ_{int} did not increase compared with the control measurement, there is an increased amount of fluctuations in the data, indicative of domain binding or induced phase separation, which are more pronounced for the LEM than the EEM. The minimal extent of interactions can be rationalized by considering the charge of the lipids comprising the model monolayers, which are either neutral or negatively charged, and the negatively charged nature of Poly(A).

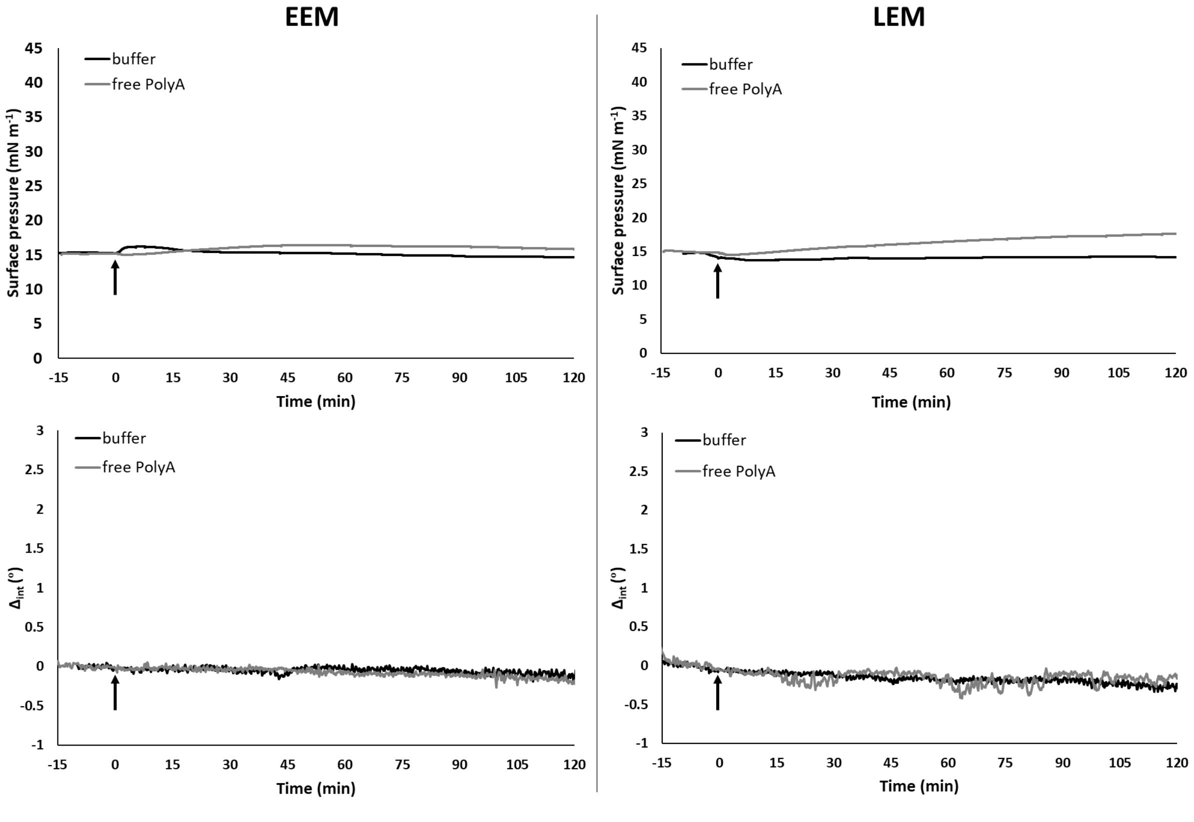

Figure S1. Change in surface pressure (top panels) and Δ_{int} (bottom panels) of EEM (left panels) and LEM (right panels) monolayers at pH 5.5 (5 mM MES buffer) after injection of 5 mM MES buffer 10% (v/v) glycerol (black lines) or free Poly(A) (1 µg mL^{-1} final concentration) (grey lines). Injection was performed at time 0 (black arrows indicate injection). The monolayers were left equilibrate before injection for 10 - 15 min.

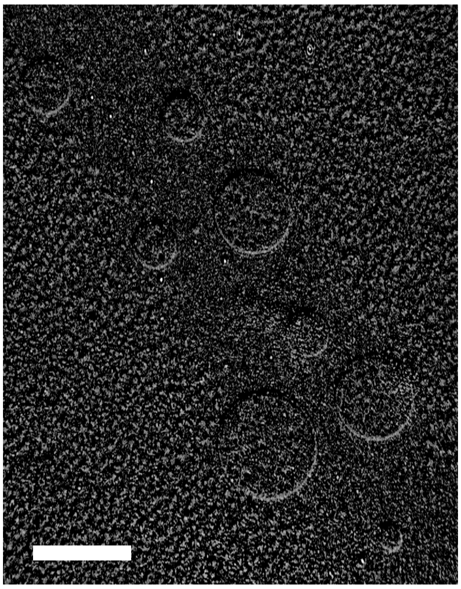

Figure S2. BAM image of models of the EEM at pH 5.5 (5 mM MES buffer) taken 75 min after injection of nucleic acid-free LNPs at a concentration equivalent to that of LNPs containing 1 µg mL^{-1} of RNA (n=2). Scale bar is 100 µm.

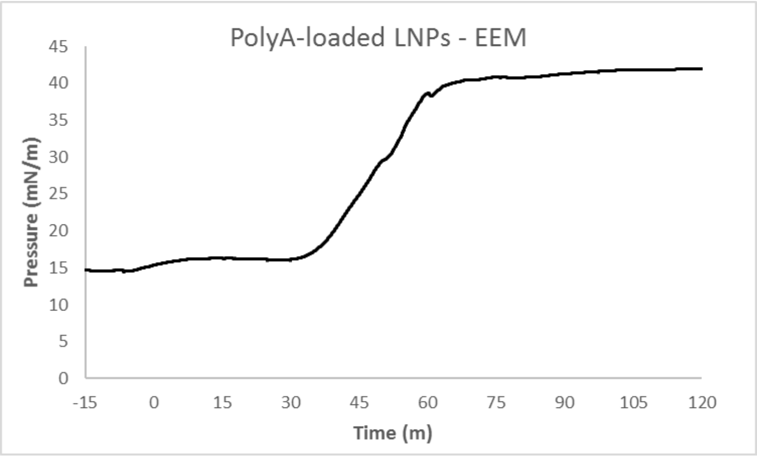

Figure S3. Change in surface pressure of EEM at pH 5.5 (PBS buffer) after injection of Poly(A)-loaded LNPs to a final RNA concentration of of 1 µg mL^{-1}. Injection was performed at time 0.

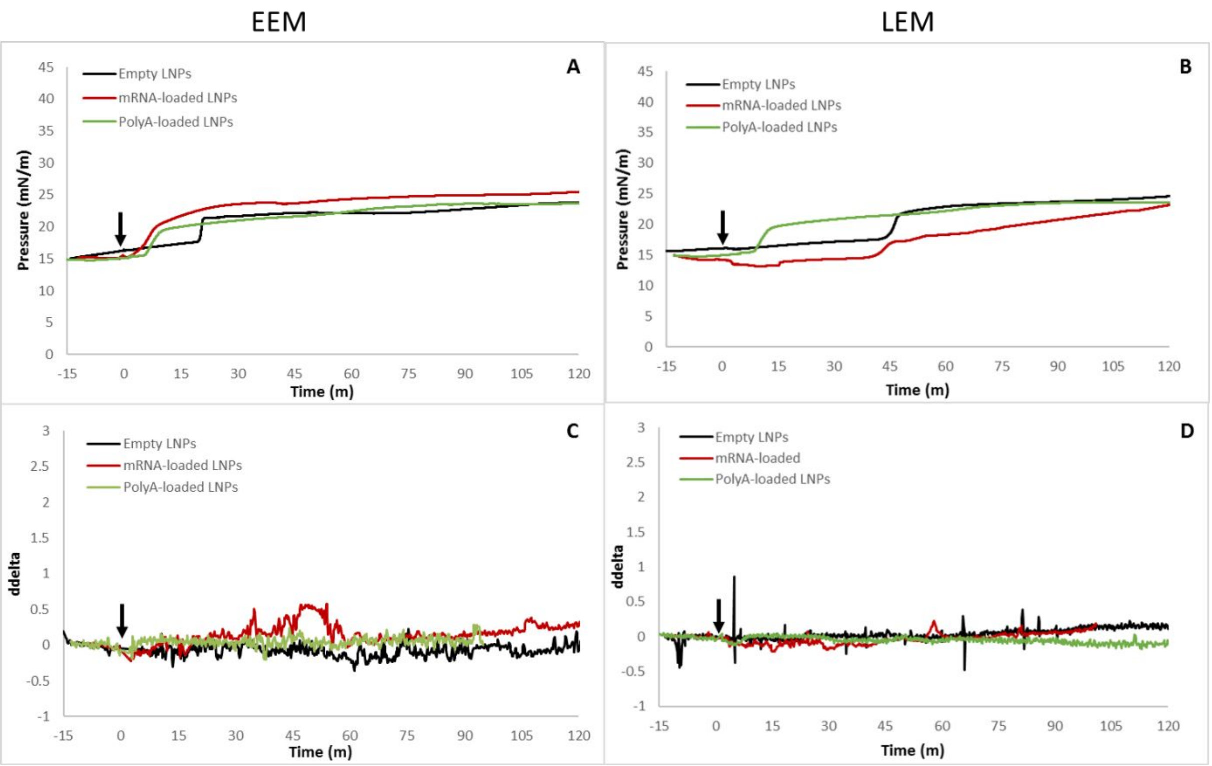

Figure S4. Change in surface pressure (A and B) and Δ_{int} (C and D) of EEM (A and C) and LEM (B and D) monolayer at pH 7.4 (PBS buffer) after injection of mRNA-loaded, Poly(A)-loaded or nucleic acid-free LNPs at a final concentration of RNA of 1 µg mL^{-1}. Injection was performed at time 0 (black arrows indicate injection). The monolayers were left equilibrate before injection for a time between 10 and 15 min.

Statistical Analysis

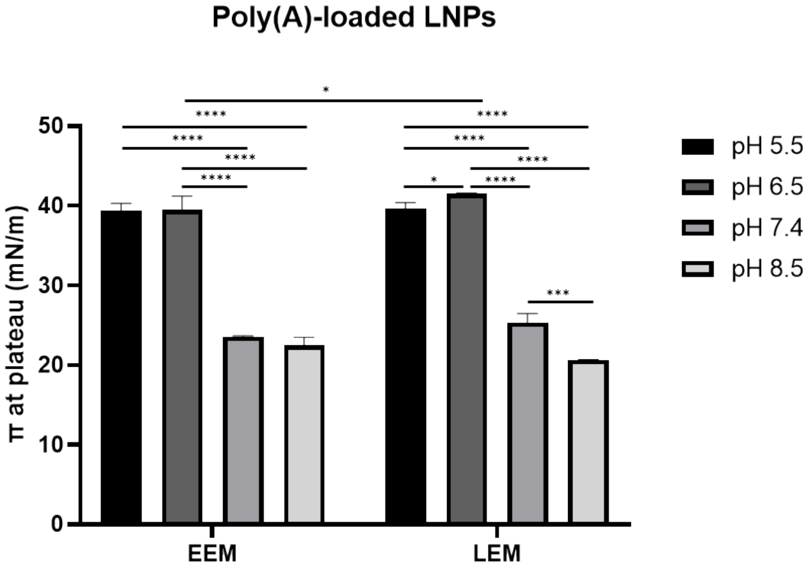

Figure S5. Final surface pressure values of EEM and LEM monolayers after injection of Poly(A)-loaded LNPs at a pH of either 5.5, 6.5, 7.4 or 8.5. π values at plateau were averaged across repeats (EEM pH 5.5 n = 6; EEM pH 6.5 n = 3; EEM pH 7.4 n = 3; EEM pH 8.5 n = 2; LEM pH 5.5 n = 5; LEM pH 6.5 n = 3; LEM pH 7.4 n = 3; LEM pH 8.5 n = 2) and plotted with ± standard deviation (SD). 2way ANOVA statistical analysis and Šídák's multiple comparisons test were performed (**** p<0.0001, *** p<0.0005, * p<0.05).

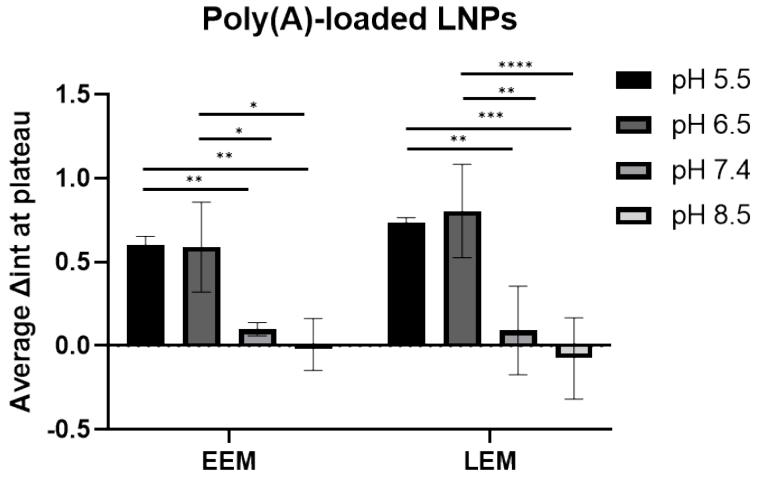

Figure S6. Final Δ_{int} values of EEM and LEM monolayers after injection of Poly(A)-loaded LNPs at pHs 5.5, 6.5, 7.4 or 8.5. The mean Δ_{int} values at plateau per each sample were averaged across repeats (EEM pH 5.5 n = 6; EEM pH 6.5 n = 3; EEM pH 7.4 n = 2; EEM pH 8.5 n = 2; LEM pH 5.5 n = 3; LEM pH 6.5 n = 3; LEM pH 7.4 n = 2; LEM pH 8.5 n = 3) and plotted with ± standard deviation (SD). 2way ANOVA statistical analysis and Šídák's multiple comparisons test was performed (**** p<0.0001, *** p<0.0005, ** p<0.005 * p<0.05).

Table S2: Comparison of empty and Poly(A)-loaded LNPs interactions with bare surface (absence of endosomal monolayers).

LNPs	Plateau reached at min	π at plateau (mN/m)	π at 150 min
**Empty**	255±24	35±2.6	23±4.9
**Poly(A)-loaded**	242±25.4	47±1.8	37.5±1

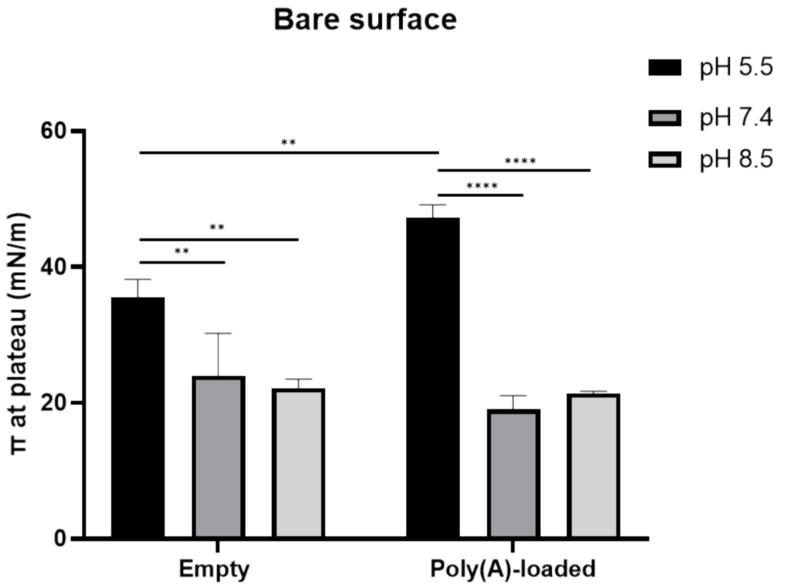

Figure S7. Final surface pressure values (at plateau) after injection of empty and Poly(A)-loaded LNPs at pHs of either 5.5, 7.4 or 8.5 with bare surface (absence of endosomal monolayers). π values at plateau were averaged across repeats (Empty pH 5.5 n = 3; Empty pH 7.4 n = 3; Empty pH 8.5 n = 3; Poly(A)-loaded pH 5.5 n = 2; Poly(A)-loaded pH 7.4 n = 3; Poly(A)-loaded pH 8.5 n = 2) and plotted with ± standard deviation (SD). 2way ANOVA statistical analysis and Šídák's or Turkey's multiple comparisons test was performed (**** p<0.0001, ** p<0.01).

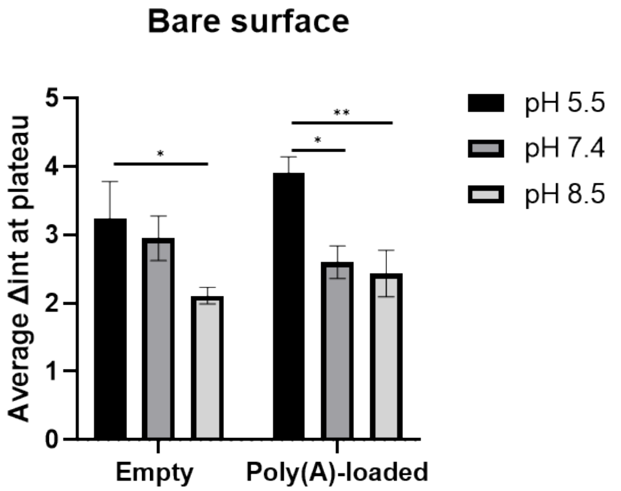

Figure S8. Final Δ_{int} values (at plateau) after injection of empty and Poly(A)-loaded LNPs at pHs of either 5.5, 7.4 or 8.5 with bare surface (absence of endosomal monolayers). The mean Δ_{int} values at plateau per each sample were averaged across repeats (Empty pH 5.5 n = 2; Empty pH 7.4 n = 2; Empty pH 8.5 n = 2; Poly(A)-loaded pH 5.5 n = 2; Poly(A)-loaded pH 7.4 n = 2; Poly(A)-loaded pH 8.5 n = 2) and plotted with ± standard deviation (SD). 2way ANOVA statistical analysis and Šídák's or Turkey's multiple comparisons test was performed (**** p<0.0001, ** p<0.01).

References

1. Arteta, M. Y.; Kjellman, T.; Bartesaghi, S.; Wallin, S.; Wu, X.; Kvist, A. J.; Dabkowska, A.; Székely, N.; Radulescu, A.; Bergenholtz, J., Successful reprogramming of cellular protein production through mRNA delivered by functionalized lipid nanoparticles. *Proceedings of the National Academy of Sciences* **2018,** *115* (15), E3351-E3360.

2. Belliveau, N. M.; Huft, J.; Lin, P. J.; Chen, S.; Leung, A. K.; Leaver, T. J.; Wild, A. W.; Lee, J. B.; Taylor, R. J.; Tam, Y. K., Microfluidic synthesis of highly potent limit-size lipid nanoparticles for in vivo delivery of siRNA. *Molecular Therapy-Nucleic Acids* **2012,** *1*, e37.

3. Chen, S.; Tam, Y. Y. C.; Lin, P. J.; Leung, A. K.; Tam, Y. K.; Cullis, P. R., Development of lipid nanoparticle formulations of siRNA for hepatocyte gene silencing following subcutaneous administration. *Journal of Controlled Release* **2014,** *196*, 106-112.

4. Ke, C.; Loksztejn, A.; Jiang, Y.; Kim, M.; Humeniuk, M.; Rabbi, M.; Marszalek, P. E., Detecting solvent-driven transitions of poly (A) to double-stranded conformations by atomic force microscopy. *Biophysical journal* **2009,** *96* (7), 2918-2925.

5. Eygeris, Y.; Patel, S.; Jozic, A.; Sahay, G., Deconvoluting Lipid Nanoparticle Structure for Messenger RNA Delivery. *Nano letters* **2020**.

6. Roces, C. B.; Lou, G.; Jain, N.; Abraham, S.; Thomas, A.; Halbert, G. W.; Perrie, Y., Manufacturing considerations for the development of lipid nanoparticles using microfluidics. *Pharmaceutics* **2020,** *12* (11), 1095.
